# The Influence of Sport Nutrition Knowledge on Body Composition and Perceptions of Dietary Requirements in Collegiate Athletes

**DOI:** 10.3390/nu13072239

**Published:** 2021-06-29

**Authors:** Andrew R. Jagim, Jennifer B. Fields, Meghan Magee, Chad Kerksick, Joel Luedke, Jacob Erickson, Margaret T. Jones

**Affiliations:** 1 Sports Medicine, Mayo Clinic Health System, La Crosse, WI 54601, USA; ckerksick@lindenwood.edu (C.K.); luedke.joel@mayo.edu (J.L.); erickson.jacob@mayo.edu (J.E.); 2 Exercise & Sport Science Department, University of Wisconsin—La Crosse, La Crosse, WI 54601, USA; 3 Patriot Performance Laboratory, Frank Pettrone Center for Sports Performance, Intercollegiate Athletics, George Mason University, Fairfax, VA 22030, USA; jfields2@springfieldcollege.edu (J.B.F.); mmagee2@gmu.edu (M.M.); mjones15@gmu.edu (M.T.J.); 4 Department of Exercise Science and Athletic Training, Springfield College, Springfield, MA 01109, USA; 5 Sport Management, George Mason University, Fairfax, VA 22030, USA; 6 Exercise and Performance Nutrition Laboratory, Lindenwood University, St. Charles, MO 63301, USA

**Keywords:** dietary intake, sport nutrition knowledge, dietary habits, energy availability

## Abstract

Sport nutrition knowledge has been shown to influence dietary habits of athletes. The purpose of the current study was to examine relationships between sport nutrition knowledge and body composition and examine potential predictors of body weight goals in collegiate athletes. Participants included National Collegiate Athletic Association Division III women (*n* = 42, height: 169.9 ± 6.9 cm; body mass: 67.1 ± 8.6 kg; fat-free mass: 51.3 ± 6.6 kg; body fat percent: 24.2 ± 5.3%) and men (*n* = 25, height: 180.8 ± 7.2 cm; body mass: 89.2 ± 20.5 kg; fat-free mass: 75.9 ± 12.2 kg; body fat percent: 13.5 ± 8.9%) athletes. Body composition was assessed via air displacement plethysmography. Athletes completed a validated questionnaire designed to assess sport nutrition knowledge and were asked questions about their perceived dietary energy and macronutrient requirements, as well as their body weight goal (i.e., lose, maintain, gain weight). Athletes answered 47.98 ± 11.29% of questions correctly on the nutrition questionnaire with no differences observed between sexes (men: 49.52 ± 11.76% vs. women: 47.03 ± 11.04%; *p* = 0.40). An inverse relationship between sport nutrition knowledge scores and body fat percentage (BF%) (*r* = −0.330; *p* = 0.008), and fat mass (*r* = −0.268; *p* = 0.032) was observed for all athletes. Fat mass (β = 0.224), BF% (β = 0.217), and body mass index (BMI) (β = 0.421) were all significant (*p* < 0.05) predictors of body weight goal in women. All athletes significantly (*p* < 0.001) underestimated daily energy (−1360 ± 610.2 kcal/day), carbohydrate (−301.6 ± 149.2 grams/day [g/day]), and fat (−41.4 ± 34.5 g/day) requirements. Division III collegiate athletes have a low level of sport nutrition knowledge, which was associated with a higher BF%. Women athletes with a higher body weight, BF% and BMI were more likely to select weight loss as a body weight goal. Athletes also significantly underestimated their energy and carbohydrate requirements based upon the demands of their sport, independent of sex.

## 1. Introduction

It is well supported that athletes have specific dietary requirements that are essential to meet the physical training demands and optimize sport performance [1,2]. Generally, athletes require higher amounts of energy, protein, and carbohydrates as a result of their higher activity levels, intensive nature of training, and increased amounts of lean body mass when compared with non-athlete populations [1,2,3,4,5]. However, previous research has indicated that athletes often fail to meet the nutritional recommendations for their respective level of training [6,7,8,9,10,11,12]. While this dietary inadequacy is likely a result of a multitude of contextual factors, one proposed reason for athletes failing to meet their nutritional requirements is a lack of sport nutrition knowledge. This nutrition knowledge gap appears to stem from a lack of understanding regarding the higher energy and macronutrient intakes required to support the physical demands of training that are specific to individual needs of athletes. Furthermore, there often appears to be a disconnect between understanding dietary strategies for optimal performance versus weight loss or physique-focused nutritional strategies, likely confounded by mainstream media and messaging on social media. Previous research has indicated athletes across all levels of competition may have a low level of nutrition knowledge [13,14,15,16,17], particularly in regard to identifying appropriate energy needs, confusion over dietary supplements, and the role of nutrition in energy production [18]. Inadequate nutrition knowledge is likely of concern to practitioners who are responsible for helping athletes optimize performance and health. Furthermore, low sport nutrition knowledge may contribute to inadequate dietary practices, which in turn may compromise an athlete’s ability to optimize performance, recovery, and health.

Previous research has demonstrated that collegiate women athletes specifically may have a misunderstanding of the advanced dietary requirements of their sport, which also appears to be accompanied by discrepancies between perceived dietary intakes and calculated dietary intakes [8,11,19]. Such a lack of sport nutrition knowledge in women athletes may be compounded with body image issues or a drive for thinness, likely underpinned by societal expectations or aesthetically influenced beliefs and ultimately conflating the issue of adequate fueling for their sport [8,20,21]. For example, Hinton et al. [8] reported that 62% of women collegiate athletes (*n* = 165) wanted to lose at least 5 lbs. compared with 23% of men collegiate athletes, which was also associated with a lower energy and macronutrient intake. There continues to be a need to understand why women athletes struggle to meet the dietary requirements of their sport and the role of sport nutrition knowledge in their nutritional choices. Otherwise, extended periods of insufficient dietary intake may predispose an athlete to undesirable changes in body composition, body mass, and performance throughout a season. Further, an athlete may be at risk of relative energy deficiency in sport (RED-s) syndrome [22] if an energy deficiency continues for extended periods of time, leading to negative effects on health and performance. Such energy deficiencies or poor dietary habits predispose an athlete to suboptimal performance, limitations in training adaptations, and a higher risk of injury or illness [22,23,24]. For example, Ihalainen et al. [24] recently reported that young elite female runners who were classified as amenorrheic (common occurrence in those with LEA) self-reported a higher number of days when they were injured (63 ± 23 days) compared with eumenorrheic (4 ± 5 days) runners throughout a year of training. Hence, it is important to ensure that athletes are aware of the nutritional requirements of their sport, based upon their level of training, while also accounting for any individualized body weight or composition goals they may have. 

Inconsistencies are present within the literature in regard to how athlete sport nutrition knowledge has been assessed [18]. In an effort to create a more standardized method of assessing sport nutrition knowledge, Trakman et al. [25,26] developed and validated a sport nutrition knowledge questionnaire, later creating an abridged version, using an online survey that can be instantaneously scored and provides immediate feedback to the athlete. A higher level of sport nutrition knowledge may positively influence athlete dietary behavior. Previous research in men professional rugby athletes indicates those with a higher sport nutrition knowledge are more likely to consume fruits, vegetables, and carbohydrate-rich foods [27]. Additionally, previous sport nutrition education interventions have been shown to improve sport nutrition knowledge [10], quality of diet [10,28], body composition and performance following the intervention [28]. It is currently unknown how sport nutrition knowledge influences body composition among collegiate athletes or what other factors may contribute to an athlete’s body weight goal. 

Within the United States, National Collegiate Athletic Association (NCAA) Division III (DIII) institutions account for ~40% of all NCAA institutions across all divisions with ~39% of all NCAA athletes competing at the DIII level. However, many DIII institutions do not have the financial resources to employ full-time sports dieticians or provide athletes with nutritional support. Therefore, the purpose of the current study was to examine the sport nutrition knowledge of NCAA Division III collegiate athletes and assess relationships between sport nutrition knowledge, body composition, and body weight goal.

## 2. Materials and Methods

### 2.1. Study Design

Prior to the start of the 2019–2020 season, athletes completed a body composition assessment, an electronic validated sport nutrition knowledge questionnaire [26,29], and an internally developed questionnaire, which examined perceived dietary requirements and body weight goals.

### 2.2. Subjects

In the current study, 67 Division III athletes (women, *n* = 42 (soccer, *n* = 27; volleyball, *n* = 8, track and field, *n* = 8); men, *n* = 25 (wrestling, *n* = 17; and football, *n* = 8)) participated (Table 1). All players were under the direction of a strength and conditioning coach and were following sport-specific training regimens with metabolic and neuromuscular demands particular to their respective sport and training program. All players were medically cleared for intercollegiate athletic participation, had the risks and benefits explained to them beforehand, signed an institutionally approved consent form to participate, and completed a medical history form. This study was conducted according to the Declaration of Helsinki guidelines and all procedures were approved by the University’s Institutional Review Board for use of human subjects in research. 

### 2.3. Procedures

#### 2.3.1. Body Composition

Body composition was assessed using air displacement plethysmography (BODPOD, Cosmed USA Inc., Concord, CA, USA) for determination of body fat percent (BF%), fat-mass (FM) and fat-free mass (FFM). At the beginning of each testing day, calibration procedures were completed according to the manufacturer guidelines, using the provided calibration cylinder of a standard volume (49.55 L). Participants were instructed to refrain from exercise, eating, and drinking for ≥4 h prior to testing. Additionally, participants were instructed to wear spandex or tight-fitting clothing, remove all jewelry, and wear a swim cap to reduce excess air displacement. Lung volume was estimated for determination of relative body volume based upon thoracic volume. Athlete body mass and body volume were used to estimate body fat composition based upon the Siri equation [30]. A trained technician performed all testing. Previous research indicated the BODPOD is a valid measure of body composition in female collegiate athletes compared with DEXA for BF% (*R*^2^ = 0.85, SEE = 2.14) [31]. Additionally, test-to-test reliability of performing this body composition assessment within our lab in athletic populations has yielded high reliability for body mass (*r* = 0.999), body fat percent (*r* = 0.994), and fat-free mass (*r* = 0.998).

#### 2.3.2. Abridged Sport Nutrition Knowledge Questionnaire

Athletes completed the Abridged Sport Nutrition Knowledge Questionnaire (A-SNKQ), which consists of 37 items that assess general (*n* = 17) and sports (*n* = 20) nutrition knowledge [26,29]. The A-SNKQ has been previously assessed for validity (construct) and reliability (test–retest) in athletes, with findings indicating a high construct validity (*p* < 0.001) and good test–retest concordance (*r* = 0.8, *p* < 0.001); therefore, it is suitable for determining sports nutrition knowledge. The questionnaire was distributed using an online electronic survey tool (Qualtrics, Provo, UT, USA), and scores from the A-SNKQ were automatically calculated upon submission. The results were interpreted as “poor” knowledge (0–49%), “average” knowledge (50–65%), “good” knowledge (66–75%) and “excellent” knowledge (75–100%) based on previously published methods [25].

#### 2.3.3. Perceived Dietary Requirements Questionnaire

Prior to the start of the season, athletes completed a brief online questionnaire (Qualtrics, Provo, UT, USA), which was internally developed (Supplementary S1) to assess perceived energy and macronutrient intake on a typical day. These responses were then compared with the calculated energy and macronutrient intake levels based on low, moderate, and high activity level recommendations provided by the International Society of Sports Nutrition (ISSN) [2,11]. The low, moderate, and high recommended daily energy intakes were calculated using a relative energy intake value of 40, 50, and 60 kcal/kg/day, respectively. Protein recommendations were calculated using relative intakes of 1.4, 1.6, and 1.8 g/kg/day. Carbohydrate recommendations were calculated using 4, 6, and 8 g/kg/day and fat recommendations were calculated from a relative percentage of total predicted energy needs, set at 15, 25, and 30%. In addition, athletes were asked to indicate their current body weight goal (i.e., lose weight, maintain weight, or gain weight), and rank their current nutritional barriers.

### 2.4. Statistical Analysis

Participant demographic data are presented using descriptive statistics by sex. Pearson correlation coefficients were used to examine relationships between sport nutrition knowledge scores, BF%, FFM, FM, body mass, and body mass index (BMI). The following criteria were used for interpreting correlation coefficients: very weak, <0.20; weak, 0.20–0.39; moderate, 0.40–0.59; strong, 0.60–0.79; and very strong, >0.80. Multinomial logistic regression was used to predict weight loss goals based on sport nutrition knowledge scores and body composition parameters. Paired sample t-tests were used to compare differences between calculated dietary requirements and perceived needs. All data were analyzed using SPSS V.25 (IBM Corporation, Armonk, NY, USA) (*p* < 0.05). Data are presented as means ± standard deviations.

## 3. Results

### 3.1. Nutrition Knowledge

Athletes answered 47.9 ± 11.3% of questions correctly on the nutrition questionnaire, with no differences observed between sexes (men, 49.5 ± 11.7%, vs. women, 47.0 ± 11.0%; *p* = 0.40). An inverse relationship between sport nutrition knowledge scores and BF% (*r* = −0.330; *p* = 0.008) and fat mass (*r* = −0.268; *p* = 0.03) was observed. No relationships between sport nutrition knowledge scores and any other body composition parameters were observed (*p* > 0.05).

### 3.2. Nutrition Knowledge and Perceived Dietary Needs

There was a positive relationship between sport nutrition knowledge scores and perceived absolute protein intake (*r* = 0.276; *p* = 0.03), absolute carbohydrate intake (*r* = 0.30; *p* = 0.027), and relative carbohydrate requirements (*r* = 0.35; *p* = 0.007) for all athletes. There was a positive relationship between sport nutrition knowledge scores and perceived energy requirements (*r* = 0.494; *p* = 0.003), relative carbohydrate requirements (*r* = 0.386; *p* = 0.020), absolute protein intake (*r* = 0.407; *p* = 0.015), and absolute carbohydrate intake (*r* = 0.343; *p* = 0.047) in women athletes. No significant relationships were observed between sport nutrition knowledge scores and any of the perceived energy and macronutrient intakes or requirements in men athletes (*p* > 0.05). All players significantly (*p* < 0.01) underestimated daily energy (−1360 ± 610.2 kcal/day), absolute carbohydrate (−301.6 ± 149.2 g/day), and absolute fat (−41.4 ± 34.5 g/day) requirements when compared with their calculated requirement, using a “moderate” activity level. A detailed summary of differences in perceived versus calculated dietary requirements for energy and macronutrient needs for men and women athletes are presented in Table 2 and Table 3, across a low, moderate, and high activity level.

### 3.3. Body Weight Goal

Of the woman athletes, 35% selected a goal of weight loss, 60.3% selected maintaining weight, and 4.8% reported a desire to gain weight. Of the men athletes, 36% reported a desire to lose weight, 56% reported a desire to maintain weight, and 8% selected a desire to gain body weight. Fat mass (β = 0.224; *p* = 0.02), BF% (β = 0.217; *p* = 0.01), and BMI (β = 0.421; *p* = 0.01) were all significant predictors of body weight goal (*p* < 0.05) in women. For every 1% increase in BF% and 1 kg increase in FM, women athletes were 1.2 times more likely to report weight loss as their current body weight goal. For every 1 unit increase in BMI, women athletes were 1.5 times more likely report weight loss as their current body weight goal. No body composition parameters were significant predictors of body weight goals in men athletes.

### 3.4. Nutritional Barriers

Across all athletes, lack of time (31.8%) and lack of knowledge (28.6%) accounted for the greatest barriers to optimal nutrition. In women players, 36% reported financial restrictions as the primary barrier, whereas 41.9% of men reported lack of time as their primary barrier (Table 4).

## 4. Discussion

The primary aim of the current study was to assess the sport nutrition knowledge of NCAA Division III collegiate athletes and examine the subsequent relationships between body composition and body weight goal. The mean number of questions answered correctly on the sport nutrition knowledge questionnaire was ~48%, which is categorized as “poor” [25]. Athletes in the current study underestimated their respective energy and carbohydrate requirements compared with their predicted needs, based on their level of training. This underestimation was more exaggerated in women athletes, who also underestimated protein and fat requirements (Table 3). Fat mass, BF%, and BMI were significant predictors of body weight goals in women, while no body composition measure was associated in body weight goals in men.

Previous studies have reported low degrees of sport nutrition knowledge using the same or similar sport nutrition knowledge questionnaires among athletes [9,13,14,18,26]. Further, the observed underestimation of energy and macronutrient requirements, particularly for daily carbohydrates, is not uncommon among athletes, specifically among women athletes [11,17,32]. Jagim et al. [11] previously assessed the perceived dietary requirements of women collegiate lacrosse players who significantly underestimated their daily energy (−1284 ± 685 kcal/day), carbohydrate (−178 ± 94 g/day), protein (−31.4 ± 29.8 g/day), and fat (−27.9 ± 18.7 g/day) requirements. Further, Jagim et al. [11] also indicated inconsistencies between the athletes’ perceived dietary intakes compared with actual intakes when using electronic food logs. Moreover, in alignment with the results from the current study, the discrepancies observed between the perceived dietary requirements versus calculated dietary requirements, indicate that collegiate athletes may not only have a poor understanding of their own dietary requirements, but also have a poor perception of what their actual intake consists of. Together, these issues are likely to further contribute to inadequate dietary practices of collegiate athletes. 

Results from the current study suggest sport nutrition knowledge is associated with certain body composition parameters, as those with a higher sport nutrition knowledge had lower BF% and FM values. These inverse relationships have been observed previously in men Australian football and soccer athletes [33]. As such, athletes with higher levels of sport nutrition knowledge may have a better understanding of how adherence to certain energy and macronutrient intakes relates to maintaining a desired body weight or composition. Interestingly, thirty-five percent of the athletes in the current study reported a desire to lose weight. Overall, there was a collective mean BF% of 24% and a mean BMI of 23.3 kg/m^2^ for the women athletes, which places them in the “fair/poor” and “normal weight” categories, respectively [34], and are also in alignment with BF% values reported among collegiate women athletes [35]. Although not assessed in the current study, body image and a drive for thinness, particularly among high-risk sports, may influence the high number of athletes reporting weight loss as a current goal or predispose women athletes to disordered eating patterns, which is a disturbing phenomenon among women’s sports [20,21,36,37]. However, in an attempt to promote healthy nutritional practices, positive body image, safe training, and appropriate body weight management practices, a current trend has seemingly emerged within the sport science community to one that focuses on optimal health within women’s sports [38]. 

Similarly, 36% of men athletes in the current study indicated a desire to lose body weight. Although, the mean body fat percentage for men was 13.5%, corresponding to a body composition rating of “good” [39], a high percentage still indicated a desire to lose weight. For team sport athletes, there is not a specified ideal BF%, but rather emphasis is generally placed upon meeting the macronutrient and micronutrient requirements, as determined from training demands, or for obtaining a weight and body composition that optimizes sport performance. There are certain circumstances of an “ideal body type” or a general understanding that a lower body weight may be advantageous for performance (i.e., running or road cycling). Additionally, weight-class athletes are limited in how much their body weight can fluctuate; therefore, a better understanding of nutritional concepts would likely allow the athlete to achieve a desired weight status more safely and efficiently.

Presumably, a higher sport nutrition knowledge would improve athlete understanding of dietary requirements and subsequently improve dietary intake, while lower degrees of sport nutrition knowledge may have the opposite effect. However, findings from previous research has been mixed in regard to the relationships between nutrition knowledge and quality of dietary intake [13]. Moreover, this relationship appears to be influenced by the level of competition and nutritional support or resources available to the athlete. Develin et al. [33] observed low sport nutrition knowledge scores in men Australian football and soccer athletes, which seemed to correspond with an insufficient intake of carbohydrates. This finding is in alignment with the relationships observed in the current study, albeit with perceived dietary intake rather than actual. Conversely, Rash et al. [40] failed to observe relationships between nutrition knowledge or attitudes toward nutrition and dietary quality in men and women collegiate track and field athletes. As described by Heaney et al. [13,41], there are likely situations in which unanticipated barriers may prevent athletes from consuming adequate amounts of energy and nutrients, despite likely knowing what their requirements are. In the current study, athletes reported a lack of knowledge and time as their biggest barriers to meeting the nutritional requirements of their level of training. Moreover, at the collegiate level, there is likely a combination of factors preventing athletes from meeting dietary requirements, such as nutrition knowledge, financial resources, lack of time, and lack of adequate facilities. However, additional research is needed to identify the primary causative factors contributing to insufficient dietary intakes of collegiate athletes. Specific attention should target the associations between nutrition knowledge, body image, societal influences, coaches’ influence, and the subsequent dietary habits of athletes.

This study is not without limitations. An individualized assessment of total daily energy expenditure would have provided a more specific indicator of the energy requirements for each athlete. Further, the lack of actual dietary-intake data limits the ability to assess energy availability or energy balance. The small sample size prevented the completion of sub-analyses to control for body composition differences or examine differences across sport groups. Further, all athletes in the current study were from a single academic institution and therefore the observed relationships may not apply to athletes competing at other institutions, particularly at higher levels of competition with more financial resources. Additionally, we did not control for prior nutrition education or coursework pertaining to exercise science and athletic performance. Prior education may influence the degree of sport nutrition knowledge and any subsequent relationships to body composition. Future research should examine the actual dietary intake for each athlete while accounting for individual measures of total daily energy expenditure to provide a more precise measure of individual energy status. This would allow for further examinations into the potential relationships between sport nutrition knowledge, dietary intake, body composition, and performance.

## 5. Conclusions

Division III collegiate athletes have a low level of sport nutrition knowledge, which was associated with a higher BF%. Women athletes with a higher body weight, BF%, and BMI were more likely to select weight loss as a body weight goal. Athletes significantly underestimated their energy and carbohydrate requirements based upon their level of training. It is recommended that sports nutrition practitioners develop practical strategies to educate athletes on basic nutritional concepts, the specific nutritional requirements of their sport, and how to apply the information in their daily routines by overcoming key barriers. Moreover, it is important to look beyond the basic energy and macronutrient recommendations as athletes may not have a clear understanding of how such recommendations translate into actual food portions or combinations needing to be consumed.

## Figures and Tables

**Table 1:** Descriptive summary of physical characteristics by sex.

Characteristic	Females (*n* = 42)	Males (*n* = 25)
Age (years)	19.5 ± 0.9	20.1 ± 1.8
Height (cm)	169.9 ± 6.9	180.8 ± 7.3
Body mass (kg)	67.1 ± 8.6	89.3 ± 20.5
Body mass index (kg/m^2^)	23.3 ± 2.7	27.1 ± 5.1
Body fat percent	24.2 ± 5.3	13.5 ± 8.9
Fat-free mass (kg)	51.3 ± 6.6	75.9 ± 12.2

Data presented as mean ± SD.

**Table 2:** Comparison of perceived dietary requirements versus predicted for men athletes.

Dietary Value	Perceived Requirements	Recommended		Delta (Perceived–Recommended)	*p* Value
Total Energy Intake (kcal/day)	3234 ± 925 (2788, 3680)	Low	3608 ± 845 (3243, 3974)	−483 ± 594 (−754, −212)	<0.001
		Moderate	4511 ± 1056 (4054, 4967)	−1386 ± 633 (−1673, −1098)	<0.001
		High	5413 ± 1268 (4865, 5961)	−2288 ± 725 (−2619, 1959)	<0.001
Relative Energy Intake (kcal/kg/day)	35.3 ± 6.3 (32.3, 38.3)	Low	40		
		Moderate	50		
		High	60		
Total CHO Intake (g/day)	216.0 ± 147.6 (145.0, 287.1)	Low	360.9 ± 84.5 (324.3, 397.5)	−151 ± 158 (−227.1, −74.9)	<0.001
		Moderate	541.3 ± 126.8 (486.5, 596.1)	−334.6 ± 176.6 (−419.7, −249.4)	<0.001
		High	721.7 ± 169.0 (648.6, 794.8)	−518.1 ± 201.6 (−615.3, −420.9)	<0.001
Relative CHO Intake (g/kg/day)	2.4 ± 1.5 (1.7, 3.1)	Low	4		
		Moderate	6		
		High	8		
Total PRO Intake (g/day)	119.8 ± 66.5 (87.7, 151.8)	Low	126.3 ± 29.6 (113.5, 139.1)	−9.4 ± 77.3 (−43.6, 24.8)	0.574
		Moderate	144.3 ± 33.8 (129.7, 159.0)	−27.7 ± 79.4 (−62.9, 7.5)	0.116
		High	162.4 ± 38.0 (145.9, 178.8)	−46.1 ± 81.7 (−82.3, −9.8)	0.015
Relative PRO Intake (g/kg/day)	1.4 ± 0.8 (1.0, 1.7)	Low	1.4		
		Moderate	1.6		
		High	1.8		
Total Fat Intake (g/day)	57.2± 38.4 (38.7, 75.7)	Low	60.1 ± 14.1 (54.1, 66.2)	−4.0± 38.9 (−22.8, 14.76)	0.659
		Moderate	100.2 ± 23.5 (90.1, 110.4)	−44.8 ± 41.8 (−64.9, −24.7)	<0.001
		High	120.3 ± 28.2 (108.1, 132.5)	−65.9 ± 43.8 (−86.3, −44.1)	<0.001
Relative Fat Intake (g/kg/day)	0.6 ± 0.4 (0.4, 0.9)	Low	15%		
		Moderate	25%		
		High	30%		

Data presented as mean ± SD with 95% confidence intervals. CHO = carbohydrates, PRO = protein.

**Table 3:** Comparison of perceived dietary requirements versus predicted for women athletes.

	Perceived Requirements	Recommended		Delta (Perceived–Recommended)	*p* Value
Total Energy Intake (kcal/day)	2181 ± 445 (2000, 2361)	Low	2720 ± 337 (2607, 2832)	−587 ± 454 (−751, −424)	<0.001
		Moderate	3400 ± 421 (3259, 3540)	−1274 ± 482 (−1447, −1100)	<0.001
		High	4080 ± 506 (3911, 4248)	−1960 ± 520 (−2149, 1773)	<0.001
Relative Energy Intake (kcal/kg/day)	32.1 ± 6.2 (29.7, 36.6)	Low	40		
		Moderate	50		
		High	60		
Total CHO Intake (g/day)	91.0 ± 90.4 (54.5, 127.5)	Low	272.0 ± 33.7 (260.7, 283.2)	−154.4 ± 130.8 (−202.4, −106.4)	<0.001
		Moderate	408.0 ± 50.6 (391.1, 424.8)	−290.7 ± 136.1 (−340.7, −240.8)	<0.001
		High	544.0 ± 67.4 (521.5, 566.4)	−427.1 ± 142.6 (−479.4, −374.8)	<0.001
Relative CHO Intake (g/kg/day)	1.4 ± 1.4 (0.8, 1.9)	Low	4		
		Moderate	6		
		High	8		
Total PRO Intake (g/day)	50.6 ± 39.8 (34.6, 66.7)	Low	95.2 ± 11.8 (91.3, 99.1)	−36.0 ± 50.6 (−53.9, −18.1)	<0.001
		Moderate	108.8 ± 13.5 (104.3, 113.3)	−49.5 ± 50.9 (−67.6, 31.5)	<0.001
		High	122.4 ± 15.2 (117.3, 127.5)	−63.1 ± 51.2 (−81.2, −44.9)	<0.001
Relative PRO Intake (g/kg/day)	0.7 ± 0.6 (0.5, 0.9)	Low	1.4		
		Moderate	1.6		
		High	1.8		
Total Fat Intake (g/day)	27.6 ± 21.2 (19.0, 36.2)	Low	45.3 ± 5.6 (43.5, 47.2)	−13.8 ± 24.5 (−22.9, −4.6)	0.004
		Moderate	75.6 ± 9.4 (72.4, 78.7)	−44.1 ± 25.4 (−53.6, −34.6)	<0.001
		High	90.7 ± 11.2 (86.9, 94.4)	−59.3 ± 26.0 (−68.9, −49.6)	<0.001
Relative Fat Intake (g/kg/d)	0.4 ± 0.3 (0.3, 0.5)	Low	15%		
		Moderate	25%		
		High	30%		

Data presented as mean ± SD with 95% confidence intervals. CHO = carbohydrates, PRO = protein.

**Table 4:** Frequency of responses for nutritional barriers.

Barrier	Women		Men		All	
	% Answered	*n*	% Answered	*n*	% Answered	*n*
Financial restrictions	36.0%	9	9.3%	4	21.0%	13
Lack of time	12.0%	3	41.9%	18	31.8%	21
Lack of knowledge	28.0%	7	23.3%	10	28.6%	18
Travel demands	0.0%	0	2.3%	1	1.6%	1
Lack of energy/motivation	0.0%	0	7.0%	3	4.8%	3
Access to food	12.0%	3	7.0%.	3	9.2	6

## Data Availability

De-identified data can be made available upon request.

## Supplementary Materials

The following are available online at https://www.mdpi.com/article/10.3390/nu13072239/s1, Supplementary S1: Athlete Body Composition, Metabolism, and Dietary Requirements Questionnaire.
