# Supplementary material for: The Influence of Sport Nutrition Knowledge on Body Composition and Perceptions of Dietary Requirements in Collegiate Athletes
Source: Nutrients. 2021 Jun 29;13(7):2239. doi: 10.3390/nu13072239 (PMC8308384; doi:10.3390/nu13072239)
Supplement: Supplementary file 1 [file nutrients-13-02239-s001.zip › nutrients-1248523-supplementary.pdf]

Appendix S1

Athlete Body Composition, Metabolism, and Dietary Requirements Questionnaire

Background Questions:

Name: ______________________ Date: __________________

Sport: ______________________ Position: ________________

Height (in): _________ Weight (lbs.): _____________

Nutrition / Body Composition Questions:

1. Have you ever taken a nutrition class (Please circle)? Yes No
2. Does the sporting organization you are part of provide you with access to nutrition information or nutritionists/dieticians?
   a. _______ Nutrition information only
   b. _______Nutrition information and access to nutritionist/dietician
   c. _______Neither of the above
3. Rank the top 3 sources of information you rely on regarding nutrition by placing a 1, 2 and 3 in the relevant boxes (1 = the source most relied upon).
   a. ____ Academic journal
   b. ____Athletic trainer / Strength & Conditioning Coach
   c. ____Coach
   d. ____Dietician
   e. ____Nutritionist
   f. ____Doctor
   g. ____Family
   h. ____Friends
   i. ____Internet search (please specific websites used) ______________________
   j. ____Mass Media (Magazine, Radio, TV)
   k. ____Social Media (Facebook, Twitter, Instagram, etc.)
   l. ____Team-mates
4. Prior to this study, have you ever used a nutrition or fitness tracking app? (Please circle)?
   Yes No
5. On a scale of 1–10 (1=worst; 10=best), how would you rate your nutrition knowledge? ________
6. What do you think your body fat percentage is (in %)? _____________
7. What do you think your resting metabolic rate is (calories/day)? ______________________
   (The amount of calories you would burn all day if you were in bed)
8. Are you trying to (please circle):
   Lose Weight Maintain Weight Gain Weight

9. How many total calories **<u>do you think you need to eat per day</u>** in order to **<u>maintain</u>** your weight? ______________

10. How many total calories **<u>do you think you need to eat per day</u>** based on your **<u>body weight goal</u>**? ______________

11. How many total calories do you think **<u>actually eat</u>** per day? ______________
12. How many grams of protein per day do you think you **<u>need to eat per day</u>**? _______________
13. How many grams of protein do you think you **<u>actually eat per day</u>**? _______________
14. How many grams of carbohydrates per day do you think you **<u>need to eat per day</u>**? ___________
15. How many grams of carbohydrates do you think you **<u>actually eat per day</u>**? _______________
16. How many grams of fat per day do you think you **<u>need to eat per day</u>**? _______________
17. How many grams of fat do you think you **<u>actually eat per day</u>**? _______________
18. Are you taking any supplements? If so, which ones and for what reason?
    - Supplement: ___________________ Reason: ________________________________
    - Supplement: ___________________ Reason: ________________________________
    - Supplement: ___________________ Reason: ________________________________
    - Supplement: ___________________ Reason: ________________________________
    - Supplement: ___________________ Reason: ________________________________
19. Where or from whom do you get most of your nutrition information from? _________________
20. Please rank the following barriers using the scale 1–6 that may prevent you from eating healthy and meet the demands of your sport (1 = Biggest Barrier, 6 = Least likely to be a barrier):
    a. ______ Financial restrictions
    b. ______ Lack of time (to grocery shop and prepare meals)
    c. ______ Lack of knowledge and information on how to eat better
    d. ______ Travel demands of the sport
    e. ______ Lack of energy/effort
    f. ______ Access to food
21. What type of support do/would you find useful, please rank from 1 (most useful) to 5 (least useful)?
    a. _____ Access to nutrition information relevant to healthy eating
    b. _____Access to nutrition information relevant to sports/training nutrition
    c. _____Access to group presentations by nutritionist/dieticians
    d. _____Individual consultations by nutritionists/dieticians'
    e. _____Cooking classes
